# Gene expression of the immunoinflammatory and immunological status of obese dogs before and after weight loss

**DOI:** 10.1371/journal.pone.0238638

**Published:** 2020-09-23

**Authors:** Thiago Henrique Annibale Vendramini, Henrique Tobaro Macedo, Andressa Rodrigues Amaral, Mariana Fragoso Rentas, Matheus Vinícius Macegoza, Rafael Vessecchi Amorim Zafalon, Vivian Pedrinelli, Lígia Garcia Mesquita, Júlio César de Carvalho Balieiro, Karina Pfrimer, Raquel Silveira Pedreira, Victor Nowosh, Cristiana Fonseca Ferreira Pontieri, Cristina de Oliveira Massoco, Marcio Antonio Brunetto

**Affiliations:** 1 Pet Nutrology Research Center, Nutrition and Production Department, School of Veterinary Medicine and Animal Science, University of Sao Paulo—USP, Pirassununga, São Paulo, Brazil; 2 Veterinary Nutrology Service, Teaching Veterinary Hospital, School of Veterinary Medicine and Animal Science, University of Sao Paulo—USP, São Paulo, São Paulo, Brazil; 3 Medical School of Ribeirão Preto, University of São Paulo—USP, Ribeirão Preto, São Paulo, Brazil; 4 Grandfood Indústria e Comércio, Dourado, São Paulo, Brazil; 5 Department of Pathology, School of Veterinary Medicine and Animal Science, University of São Paulo–USP, São Paulo, São Paulo, Brazil; Ehime University Graduate School of Medicine, JAPAN

## Abstract

Obesity is characterized by a low degree of chronic inflammation state that, along with metabolic modifications, promotes important changes in the animal’s organism. Adipose tissue actively participates in inflammation and immunity, and several defense cells of the organism may, therefore, be involved in the diversity found between obese and ideal weight individuals. Studies regarding this subject have shown immune cell changes in humans and rats, however, the literature is scarce in relation to dogs. Thus, the present study aimed to evaluate the gene expression profile of immunoinflammatory response and the lymphoproliferation of obese dogs before and after weight loss. Eight female dogs, neutered, of different breeds, aged between 1 and 8 years (4.74±3.19), obese, with body condition score (BCS) of 9 out of a 9-point scale and body composition determined by the deuterium isotope dilution method were included. The obese dogs were enrolled in a weight loss program and after losing 20% of their initial weight became a second experimental group. A third experimental group consisted of eight female dogs, neutered, aged between 1 and 8 years (3.11±0.78) and with ideal BCS (5 out of a 9-point scale). Gene expression of immunoinflammatory cytokines (resistin, leptin, adiponectin, TNF-α, IL-6, IL-8, and IL-10) was assessed by qRT-PCR and immunity was assessed by lymphoproliferative response using the flow cytometry technique. The data that presented normal distribution was evaluated by analysis of variance by the PROC MIXED of the SAS and when differences were detected, these were compared by the Tukey test. Regarding the gene expression data, the procedure PROC GLIMMIX was adopted and the methodology of generalized linear model was used, in which the Gama distribution proved to be adequate. Values of p<0.05 were considered significant. The mean weight loss period of the animals included in the study was 194.25 ± 28.31 days and the mean weekly weight loss rate was 1.02 ± 0.82%. The average fat mass, both in percentage (P<0.001) and in kilograms (P = 0.012), was higher in the obese group (40.88%; 8.91kg), returning to normal and without difference between the control group (19.16%; 3.01kg) and after weight loss (22.10%; 4.11kg). The weight loss program resulted in an increase in percentage of lean body mass (P = 0.001), 55.50% in obese animals vs 77.90% in obese dogs after weight loss, the latter with no difference when compared to the control group (80.84%). The obese group presented increased gene expression of resistin and IL-8 in relation to the weight loss group (P = 0.002). In adiponectin, the obese group presented increased mRNA gene expression when compared to the weight loss group (P = 0.003). The evaluation of lymphocyte proliferation showed differences between the group of obese animals before and after weight loss (P = 0.004). Weight loss resulted in an increase in the lymphoproliferation rate (18.48%) compared to obese dogs at the beginning of the study (10.71%). These results indicate that weight loss modulates the immunoinflammatory response of obese dogs and may present important benefits to health and longevity of dogs.

## Introduction

Obesity is defined by the World Health Organization (2017) [1] as an abnormal or excessive fat accumulation that can impair well-being and health. The National Institute of Health (1985) [2] also classifies obesity as an energy accumulation in the form of body fat, enough to contribute as a disease. As for pets, Laflamme (2012) [3] classified obesity as a clinical syndrome resulting in the excess of body fat sufficient to compromise health and function of different organs and systems. Therefore, it is possible to state that all definitions point to the relation of obesity with health impairment, independent of species.

The relationship between owners and pets has gained importance and has overcome simple ownership and become a relation that is ruled by affection. The increased bonding between humans and animals can increase the value of healthcare and pet well-being. On the other hand, in many cases it can result in over-humanization of pets, mirroring some behaviors and diseases of humans. Obesity, therefore, becomes the main nutritional disease not only in humans but also in pets [3].

Some studies evaluated the frequency of obesity. Gossellin et al. (2007) [4] described frequencies between 20 and 40% in dogs of the general population. In another study that included 21,754 dogs from the United States, it was observed that 29.0% of the population was overweight and 5.1% was obese [5]. In Australia, 41% of pets were considered overweight [6] and in Europe, these frequencies vary from 24% to 41% of dogs that are overweight or obese [7–9]. A study conducted in China with 2,391 dogs observed that 44% of them were obese [10], and a recent study in Japan observed that 39.8% of the studied population was considered overweight and 15.1% was considered obese [11]. In Brazil, preliminary data from our research group point to a prevalence of around 41% of overweight and obese dogs in the city of Sao Paulo [12].

The consequences of obesity on the health of dogs and cats are widely described in the veterinary literature, and include reduced lifespan [13], as well as orthopedic [14–16], cardiovascular [7, 17–20], respiratory [21–24] and metabolic disorders, such as insulin resistance [25] and hyperlipidemia [26–28].

However, research regarding the metabolic properties of adipose tissue and its capacity to produce hormones with roles on physiological and physiopathological pathways modified the concept of adipocyte biology [29–32]. The increase of body fat mass has been associated with metabolic alterations that may lead to the increase of risk for developing diseases and decrease of lifespan in dogs, cats, and humans [25, 33–35]. These metabolic alterations include the production and secretion of various pro-inflammatory substances that are associated with chronic low-grade inflammation, which is characterized by deregulation of a net of inflammation signaling pathways, abnormal cytokine production, like adipokines, and increase in circulating acute-phase proteins [36]. These adipokines are currently being studied as an important connection between obesity and immune disorders [37, 38].

Chronic low-grade inflammation is one of the causes of these metabolic disorders [39]. Milner and Beck (2012) [40] suggest that the immune processes involved in the organism’s defense are affected by nutritional status and thus both incidence and severity of various diseases are increased in obese humans when compared to humans with ideal body condition. In the same study, authors observed decreased lymphocytic immune response in obsess individuals, which may predispose to infections, decreased vaccine response and insufficient wound healing.

Animal models that mimic altered human immune responses caused by obesity are currently limited to rodents, in which obesity can be naturally acquired or induced in a shorter period of time. Dogs, however, are more and more becoming an object of study regarding these metabolic alterations because they present a strong genetic homology, which makes this species a potential model for studies of human obesity [41].

Given the importance of obesity in both pets and humans, the present study aimed to evaluate the link between obesity and the immune system in dogs and the changes in the immune system after weight loss.

## Materials and methods

The experimental procedures were approved by the Animal Use Ethics Committee from the School of Veterinary and Animal Science of the University of Sao Paulo (protocol 4668091214). All owners of the participating animals gave written consent.

### Animals and treatment

Eight female neutered dogs of different breeds (Yorkshire Terrier = 1, Golden Retirever = 1, Teckel = 1, Border Collie = 1, Pinscher = 1, mixed breed = 3) were included, with a mean (± standard deviation) age of 4.74±3.19 years. Dogs were selected from the teaching veterinary hospital of the School of Animal Science and Food Engineering of the University of Sao Paulo, as well as the Pet Nutrology Research Center (CEPEN pet) of the School of Veterinary Medicine and Animal Science of the University of Sao Paulo. All animals had acquired obesity for more than 2 years, with body condition score (BCS) of 9 on a 9-point scale according to Laflamme (1997) [42]. Their body composition was determined by the method of deuterium isotopes dilution as described by Ferrier et al. (2002) [43] and Brunetto et al. (2011) [25]. After evaluation of the body composition, gene expression of immunoinflammatory cytokines, and lymphoproliferative response, the same dogs were enrolled in a weight loss program and then composed a new experimental group after loss of 20% of body weight. A third experimental group was composed of eight healthy dogs of different breeds (Border Collie = 1, West Higlhland White Terrier = 1, Siberian Husky = 1, mixed breed = 5), neutered females, mean (± standard deviation) age 3.11±0.78 years, and with ideal (5) BCS.

Dogs initially underwent a complete physical examination, nutritional evaluation, BCS and muscle mass score (MMS) assessment [44], complete blood count, and biochemical profile (albumin, glucose, total protein, urea, creatinine, alkaline phosphatase, cholesterol, triglycerides, aspartate aminotransferase, and alanine aminotransferase).

All dogs that were included in the present study presented physical and laboratory parameters within the normal range for the species, and that did not present any comorbidities, such as cardiovascular, respiratory, orthopedic, hepatic, renal, and endocrine disorders. Dogs were excluded if owners were not willing to participate in the weight loss program according to the protocol of the study.

### Complete blood count and biochemical profile

Blood samples were collected after 12 hours of fasting from the jugular, cephalic or saphenous veins. For the complete blood count, samples of 4ml were placed in EDTA tubes. All other biochemical exams were performed with serum samples, 4ml, collected in tubes without anticoagulants, centrifuged at 3000 rpm for a period of 10 minutes (Novatécnica, Piracicaba, Brazil). For glucose measurement, 3ml samples were placed in tubes containing sodium fluoride.

Complete blood counts were performed by the Lab Animal laboratory (Leme, Sao Paulo, Brazil) in an electronic counter (ABC Vet, Horiba, Brazil). Hematocrits were determined by the use of microcapillary tubes and differential leukocyte count was performed by optic microscopy of blood smear with panoptic coloring.

All biochemistry analyses were performed by the Multiuser Laboratory of Animal Nutrition and Bromatology of the Department of Animal Nutrition and Production of the School of Veterinary Medicine and Animal Science of the University of Sao Paulo. Serum creatinine, total proteins, albumin and aspartate aminotransferase were analyzed with commercial kits from Labtest (Lagoa Santa, Brazil); alanine aminotransferase, alkaline phosphatase, cholesterol, and triglycerides were analyzed with a commercial kit from Biosystems (Barcelona, Spain); and serum urea was analyzed with a commercial kit from Diasys (Holzheim, Germany).

### Adaptation period

Both the obese and healthy groups received a commercial dry diet for adult dogs (Golden Formula, Grandfood Industry, Dourado, Brazil) for 28 days for a standardized diet for initial evaluation, which composition is presented in Table 1.

**Table 1:** Composition of the control diet and its ingredients^1^.

Item	%	unit / kg	unit / 1000 kcal of ME^2^
Moisture	7.96	79.6g	26.7g
Crude protein	25.51	255.1g	85.6g
Ether extract	12.60	126.0g	42.3g
Ash	5.30	53.0g	17.8g
Crude fiber	1.91	19.1g	6.4g
Calcium	1.13	11.3g	3.7g
Phosphorus	0.85	8.5g	2.8g
Metabolizable energy	-	3795kcal	1000kcal

^1^Bovine meat meal, poultry by-product meal, isolated pork protein, whole-grain cornmeal, broken rice, beet pulp, degreased rice bran, poultry fat, pork fat, flaxseed, hydrolized pork, hydrolyzed poultry, propionic acid, BHA, BHT, potassium chloride, sodium chloride, dry brewers yeast, yeast cell wall, vitamin A, vitamin B12, vitamin C, vitamin D3, vitamin E, vitamin K3, folic acid, pantothenic acid, biotin, choline chloride, niacin, pyridoxine, riboflavin, thiamin, potassium iodine, selenium, copper sulfate, iron sulfate, manganese sulfate, zinc sulfate.

Maintenance energy requirement (MER) for each dog was calculated according to the equation 95 x (body weight)^0,75^ = kcal/day [45, 46], and the daily amount of food intake was determined by dividing the MER by the metabolizable energy of the diets.

### Body composition

The body composition of the dogs was determined by the method of deuterium isotopes dilution. The dogs were fasted for 8 hours and water fasted for 2 hours, then 1ml/kg body weight of deuterium oxide at 10% solution was administered subcutaneously. Blood samples of 3ml were obtained by jugular venopunction before and after 2 hours of deuterium oxide administration. Blood samples were processed for serum extraction and serum was stored at -20°C until analysis.

Sample deuterium enrichment was determined by isotopic ratio mass spectrometry (Calixto System, Sercon Ltd, Gateway, United Kingdom) at the Isotopic Ratio Mass Spectrometry Laboratory of the Internal Medicine Department of the Medical School of Ribeirao Preto of the University of Sao Paulo. Body composition was then determined according to the methodology described by Ferrier et al. (2002) [43] and Brunetto et al. (2011) [25].

After quantification of total body water, the total lean mass was calculated, and the percentage of fat mass was calculated by difference. This procedure was performed in the obese group before and after weight loss, and at one time point in the control group.

### Gene expression of the immune-inflammatory response

Gene expression of the cytokines was evaluated by quantitative real-time polymerase chain reaction (qRT-PCR), according to Tamura et al. (2014) [47]. Evaluated reference cytokines and genes were: resistin, leptin, adiponectin, TNF-α, IL-6, IL-8, IL-10 and glyceraldehyde-3-phosphate dehydrogenase (GAPDH).

Blood samples of 5ml were collected from cephalic, jugular or saphenous veins. They were placed in EDTA tubes, and in maximum 20 minutes after collection ratios of 250μL of blood and 750μL of TRIzol™ LS Reagent (Thermo Fisher Scientific–Invitrogen, Carlsbad, USA) were placed in a cryotube, which were put in -80°C until analysis at the Laboratory of Pharmacology and Applied Toxicology of the School of Veterinary Medicine and Animal Science of the University of Sao Paulo. Samples were then thawed and centrifuged for 5 minutes at 1200g at 4°C, as the protocol described by Tamura et al. (2014) [47]. After centrifuging, 1,5ml of supernatant was transferred to a microtube with 400μL of chloroform and was incubated for 2 to 3 minutes. The samples were then centrifuged for 15 minutes at 1200g at 4°C and, afterward, the aqueous phase was submitted to total RNA extraction by the RNeasy Mini Kit (Qiagen, Hilden, Germany) according to the manufacturer’s instructions. Total RNA concentrations were quantified by spectrophotometry reading in Nanodrop at 260nm, and RNA amount was verified by the DO 260nm/280nm ratio.

For the cDNA synthesis with the Superscript enzyme (Life Technologies, Carlsbad, USA), 100ng of total RNA from each sample was used. To each sample, 1μL of Oligo dT at 50μM (Life Technologies, Carlsbad, USA) and 4μL of dNTP Mix at 10mM (Life Technologies, Carlsbad, USA) were added, and DEPC-diethylpyrocarbonate treated water was used to reach 20μL of solution. It was then incubated for 5 minutes at 65°C and 1 minute at 4°C. Samples were amplified according to the cycle: initial denaturation at 95°C for 60 seconds, followed by 42 cycles of denaturation at 95°C for 15 seconds and extension at 60°C for 1 minute. Each sample was evaluated in triplicate for each gene. Every qRT-PCR reaction contained a negative control.

The quantification of cytokine expression levels was performed by qRT-PCR and monitored by the incorporation method of SYBR Green (SYBR Select Master Mix–Applied Biosystems, Foster City, USA) with specific primers (Table 2).

**Table 2:** Primer sequences used to detect gene expression of adipokines and housekeeping of dogs enrolled in the present study.

Gene	5`to 3`primer sequence	Amplicon size (pb)	n. of access to GenBank	Reference
Resistin	**F**^1^ –ACAGAACCTGGGAGTTGGTG	167	XM_005632937.2	Designed and tested in this study
	**R**^2^ –GGAAGCCGTGATACCAAGAA			
Adiponectin	**F**^1^ –AAGGAGATCCAGGTCTTGTTGG	416	LT963133.1	EISELE et al. (2005) [49]
	**R**^2^ –TTCCAGATGAAGGAGCACAGAG			
Leptin	**F**^1^ –CTGTGCCAATCCGAAAAGTC	387	NM_001003070.1	EISELE et al. (2005) [49]
	**R**^2^ –GTCTGTTCAGAGCCACCACC			
Interleukin - 8/CXCL8	**F**^1^ –CTCTCTGTGAAGCTGCAGTTCTG	81	NM_001003200	TAMURA et al. (2014) [47]
	**R**^2^ –GGAAAGGTGTGGAGTGTGTTTTT			
Interleukin -10	**F**^1^ –CGGGAGGGTGAAGACTTTCT	144	NM_001003077	TAMURA et al. (2014) [47]
	**R**^2^ –GGCATCACCTCCTCCAAGTA			
Interleukin - 6	**F**^1^ –TTAAGTACATCCTCGGCAAAATCT	86	NM_001003301	TAMURA et al. (2014) [47]
	**R**^2^ –CAGTGCCTCTTTGCTGTCTTCA			
Tumoral necrosis factor alfa	**F**^1^ –TCTCGAACCCCAAGTGACAAG	86	NM_001003301	TAMURA et al. (2014) [47]
	**R**^2^ –CAACCCATCTGACGGCACTA			
Glyceraldehyde-3-phosphate dehydrogenase	**F**^1^ –GCCTTGGATCTCTTGATGGA	91	NM_001003142.2	TAMURA et al. (2014) [47]
	**R**^2^ –TTCTTGGCTCTTATGCGATG			

^1^ Forward;

^2^ Reverse.

Since most primers were designed for PCR and not RT-qPCR, efficiency of primers was confirmed by dilution of a control cDNA sample and construction of a standard curve. Efficiency of at least 80% was detected for all primers used.

The cDNAs were diluted 10 times for amplification of the described gene products. Their quantification was performed by real-time PCR Step One Plus® (Applied Biosystems, Foster City, USA) in triplicate. Confirmation of the amplification products was done by analysis of dissociation curve. GAPDH gene was used as a reference gene for qRT-PCR data analysis and the method used for gene expression with qRT-PCR assay was Delta-Delta Ct [48].

### Lymphoproliferation assay

Blood samples of 5ml were collected from cephalic, jugular or saphenous veins. They were placed in tubes with lithium heparin and taken for isolated peripheral blood mononuclear cells (PBMC) using Ficoll-Histopaque 1077 gradient centrifugation at Laboratory of Bioinformatics and Genomics Applied to Veterinary Medicine of the Department of Animal Nutrition and Production of the School of Veterinary Medicine and Animal Science of the University of Sao Paulo.

Blood samples were diluted in conic sterile 15ml Falcon centrifuge tubes, in a ratio of 1ml of blood to 3ml of sterile saline phosphate buffer (PBS). Afterward, Ficoll-Paque Plus (GE Healthcare, Illinois, USA) was added to each tube in a ratio of 1:2 Ficoll to diluted blood. Samples were centrifuged for 25 minutes at 900g at 20°C without break, and then the lymphocyte band was placed in another conic sterile 15ml Falcon centrifuge tube with 10ml of sterile PBS and centrifuged once again for 5 minutes at 300g at 8°C. After the centrifuge process the supernatant material was removed and the tube was again centrifuged for 5 minutes at 300g at 8°C.

Afterward, the supernatant was again removed and 900μL of bovine fetal serum (Sigma-Aldrich, Missouri, USA) and 100μL of Hybri-Max™ dimethyl sulfoxide (DMSO) (Sigma-Aldrich, Missouri, USA) was added to the remaining solution. The sample was then placed in a cryogenic tube and put in an -80°C freezer until analysis by the Applied Pharmacology and Toxicology Laboratory of the School of Veterinary Medicine and Animal Science of the University of Sao Paulo.

**Fig 1:**
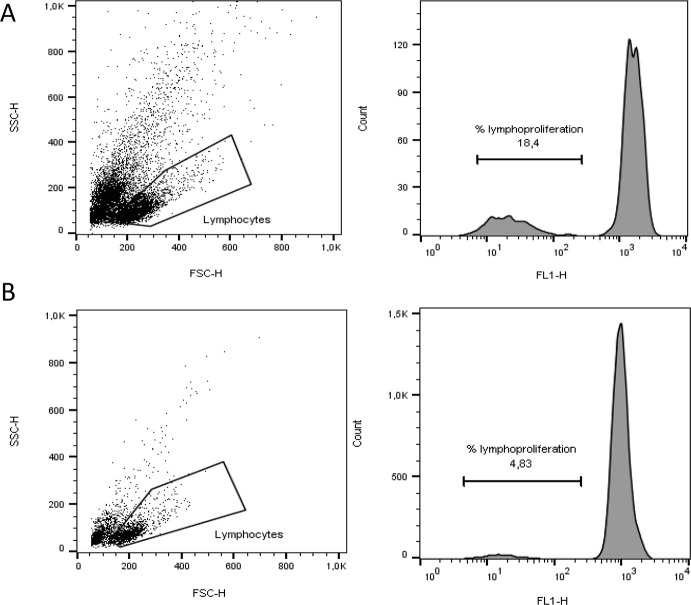
Representative flow plots of gating strategy to determine lymphoproliferation index. The gate includes lymphocyte populations based on forward and side scatter (FSC and SSC). Following, the CFSE assay proliferation indices were calculated as a ratio of the percentage of cells proliferating after mitogen stimulation (PHA) (A) to the percentage of cells proliferating without stimulation (B), according to fluorescence histogram.

The lymphoproliferation assay was performed through dividing cell tracking (DCT) technique utilizing CFSE staining were the decrease of CFSE fluorescence in filial generations allows tracking of cell divisions. Twaned lymphocytes at 1x10^7^cells/ml were labeled with carboxyfluorescein succinimidyl ester (CFSE- final concentration of 5μmol/L in PBS) for 20 minutes at 37°C and protected from light. After labeling, cells were incubated for 5 minutes in dark at 20°C, and then, 10ml of RPMI with 5% FBS (fetal bovine serum, Sigma-Aldrich, Missouri, USA) was added and centrifuged at 300 g for 5 minutes at 20°C. Cells were washed three times and 1ml RPMI per 5% FBS was added. The cells were plated in a 96-well U bottom plate at a concentration of 2x10^5^ cells/well in triplicate with either medium alone and stimulated with 1μg per 100μL PHA-L (phytohemagglutinin-L, Sigma-Aldrich, Missouri, USA) and plate was incubated for 72 hours at 37°C in a 5% CO_2_ atmosphere. At the end of this period, the cells were collected and the acquisition of the events was performed on FACSCalibur flow cytometer (Becton & Dickinson). For each sample, at least 30.000 signals were analyzed and during data acquisition a gate was drawn to select lymphocytes which were identified based on cell characteristic properties in the forward (FSC) and side (SSC) scatter than a histogram of green fluorescence (FL-1 channel) was defined within the lymphocyte cell gate. The analysis of PHA-stimulated cells within gated cells were used to calculate the percentage of T cells that had moved from the resting to the blast population and the proliferation index (PI) of mitotic cells was calculated using FlowJo cell cycle analysis software (BD Biosciences, USA) calculated as a ratio of the percentage of cells proliferating after mitogen stimulation to the percentage of cells proliferating without stimulation. A representative figure describes the analysis strategy of gate and fluorescence histogram (Fig 1).

### Weight loss protocol

To accomplish weight loss, obese dogs were fed to meet the weight loss energy requirement (WLER) [25] estimated for target weight (TW) according to the equation: WLER = 70 x (TW)^0.75^ kcal/day. Target weight was considered as the initial body weight minus 20% [24, 25, 50].

In addition, in order to avoid excessive loss and even the possibility of loss of muscle mass, using excess or lost weight, insufficient weight loss that does not guarantee the effectiveness of the program; was calculated the weekly weight loss rate (WWLR) minimum (WWLR minimum) and WWLR maximum (WWLR maximum) through the equations: minimum WWLR (g) = Current Body Weight (kg) x 10; and, WWLR maximum (g) = Current Body Weight (kg) x 20 [24, 25, 50].

The daily food amount was determined according to WLER and metabolizable energy of the diet used in the study (Premier Clinical Nutrition Canine Obesity, Grandfood Ind., Dourado, Brazil). Guaranteed analysis and ingredients of the extruded diet are described in Table 3.

**Table 3:** Guaranteed analysis and ingredients of the weight loss diet of the present study.

Item	%	unit / kg diet	unit / 1000 kcal of ME^2^
Moisture	8.14	81.4g	27.3g
Crude protein	36.92	369.2g	123.9g
Crude fat	10.27	102.7g	34.5g
Ash	5.61	56.1g	18.8g
Crude fiber	10.37	103.7g	34.8g
Calcium	0.98	9.8g	3.3g
Phosphorus	0.79	7.9g	2.6g
Metabolizable energy	-	2979kcal	1000kcal

^1^Poultry by-product meal, wheat gluten, isolated pork protein, powdered pork plasma, dehydrated egg, pea meal, barley, broken rice, cellulose, beet pulp, poultry fat, fish oil, hydrolyzed poultry and pork, propionic acid, β-glucan, potassium chloride, sodium chloride, dry brewer’s yeast, yeast cell wall, taurine, vitamin A, vitamin B_12_, vitamin C, vitamin D_3_, vitamin E, vitamin K_3_, folic acid, pantothenic acid, biotin, choline, niacin, pyridoxine, riboflavin, thiamin, chelated iron, potassium iodate, chelated manganese, selenium, copper sulphate, iron sulphate, zinc sulphate, manganese sulphate, chelated zinc, chelated copper.

The daily food amount was offered by owners in two or three meals a day. Animal assessment, WWLR, and food readjustments were made every 15 days. Owners received a recommendation to exercise their dogs for at least 15 minutes three times a week.

### Statistical analysis

Data that presented normal distribution were submitted to variance analysis at 5% significance levels with PROC MIXED from Statistical Analysis System version 9.3 (SAS, 1995), and when the difference between means was detected they were compared by Tukey test.

Statistical analysis of data regarding gene expression was performed with PROC GLIMMIX from Statistical Analysis System version 9.4 (SAS, 1995). Statistical model contemplated fixed effect between groups (obese before vs obese after weight loss) and random residual effect. For these variables, a generalized linear model methodology was used, assuming Poisson, negative binomial or gamma distributions. For all distributions, logarithmic link functions were used. Gama distribution proved to be adequate based on graphic analysis of marginal residues and by AIC criteria according to Akaike (1974) [51].

## Results

**Fig 2:**
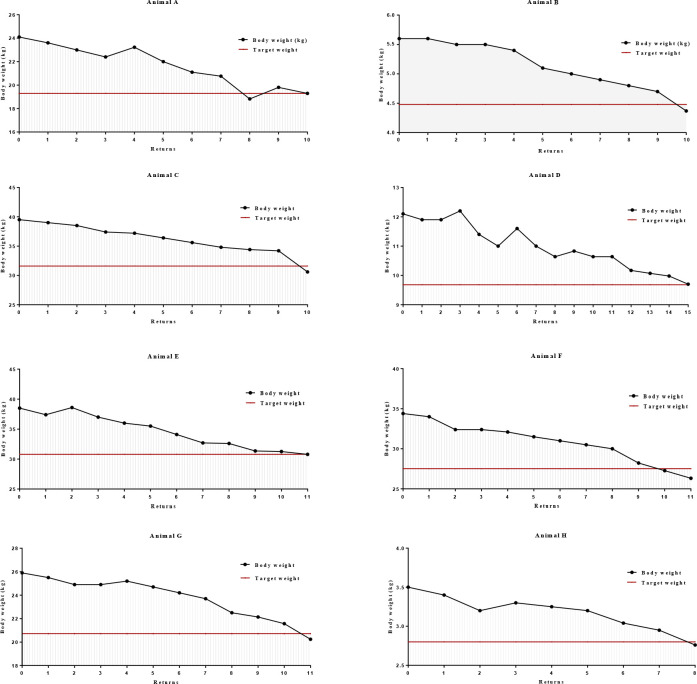
Weight loss curves throughout the weight loss program of each obese animal.

The average weight loss period of the animals included in the study was 194.25 ± 28.31 days and the average weekly weight loss rate was 1.02 ± 0.82%. Fig 2 illustrates the weight loss curve throughout the weight loss program of each animals included in the study, respectively from animal A to animal H. In addition, the figure further illustrate the proposed target weight for each animal in red.

Concentrations of alanine aminotransferase (ALT) were higher in obese dogs after weight loss when compared to the control group (P = 0.032). There were no other differences regarding biochemical analysis (Table 4).

**Table 4:** Biochemistry exams of the control group and obese group before and after weight loss.

Variable	Obese	Control	After weight loss	SEM	P
Albumin (g/dL)	3.26	3.19	2.92	0.134	0.832
Glucose (mg/dL)	88.64	90.63	88.63	5.368	0.578
Total protein (g/dL)	6.16	6.67	7.07	0.974	0.632
Urea (mg/dL)	28.67	35.38	41.1	3.077	0.324
Creatinine (mg/dL)	1.22	1.19	0.93	0.459	0.486
Alkaline phosphatase (UI/L)	49.73	25.49	20.69	9.814	0.458
Cholesterol (mg/dL)	163.89	215.21	196.22	11.608	0.194
Triglycerides (mg/dL)	94.07	56.32	57.20	11.424	0.321
AST^1^ (UI/L)	15.28	13.25	17.35	1.299	0.453
ALT^2^ (UI/L)	15.74^AB^	14.11^B^	25.06^A^	1.914	0.032

A, B - Averages followed by the same letter in the lines do not differ from each other, as determined by Student’s t-test (p < 0.05).

^1^Aspartate aminotransferase;

^2^Alanin aminotransferase

Animals from the obese group before weight loss presented higher body condition scores than animals from the control group (P < 0.001). There was no difference between the control group and the obese group after weight loss (Table 5). Mean fat mass percentage and weight were higher in the obese group before weight loss (P <0.001 and P = 0.012, respectively), but after weight loss there was no difference when compared to the control group.

**Table 5:** Weight, body condition score and body composition of the control group and the obese group before and after weight loss.

Variable	Obese	Control	After Weight Loss	SEM	P
BCS^1^	9.00^A^	5.00^B^	5.75^B^	0.383	<0.001
Body weight (kg)	22.81	16.06	18.04	2.380	0.515
Fat mass (kg)	8.91^A^	3.01^B^	4.11^B^	0.914	0.012
Lean mass (kg)	18.75	13.04	13.92	1.841	0.410
Fat mass (%)	40.88^A^	19.16^B^	22.10^B^	2.601	<0.001
Lean mass (%)	55.50^B^	80.84^A^	77.90^A^	3.503	0.001

A, B - Averages followed by the same letter in the lines do not differ from each other, as determined by Student’s t-test (p < 0.05).

^1^ Body condition score.

**Fig 3:**
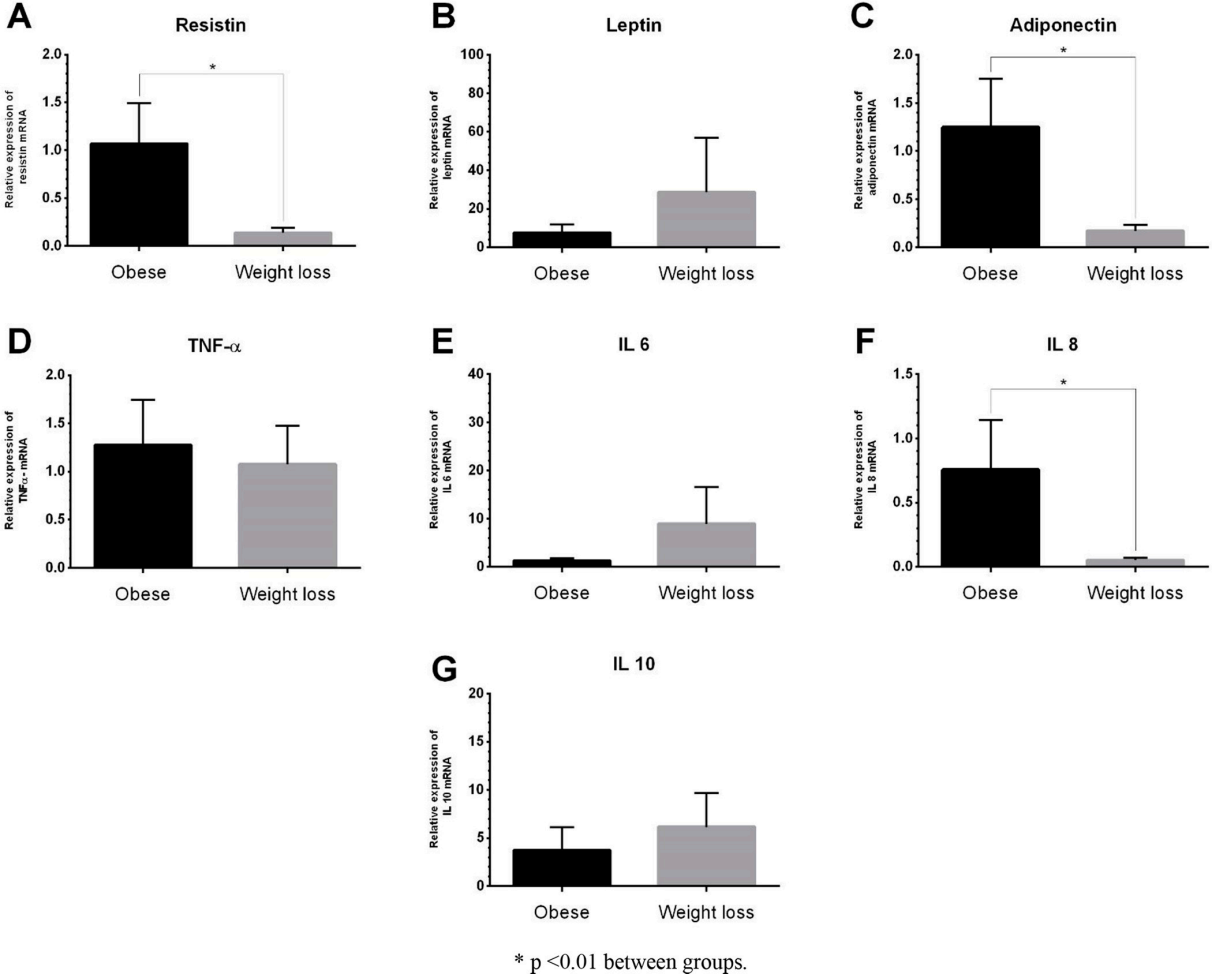
Relative expression of mRNA of resistin (A), leptin (B), adiponectin (C), tumoral necrosis factor alfa (D), interleukin 6 (E), interleukin 8 (F), and interleukin 10 (G) in total blood of obese dogs before and after weight loss.

No differences were observed in the mRNA gene expression of leptin, TNF-α, IL-6, and IL-10 of obese dogs before and after weight loss. The obese group before weight loss presented increased expression of resistin (P = 0.002), adiponectin (P = 0.003), and IL-8 (P = 0.002) when compared to the obese group after weight loss (Fig 3).

**Fig 4:**
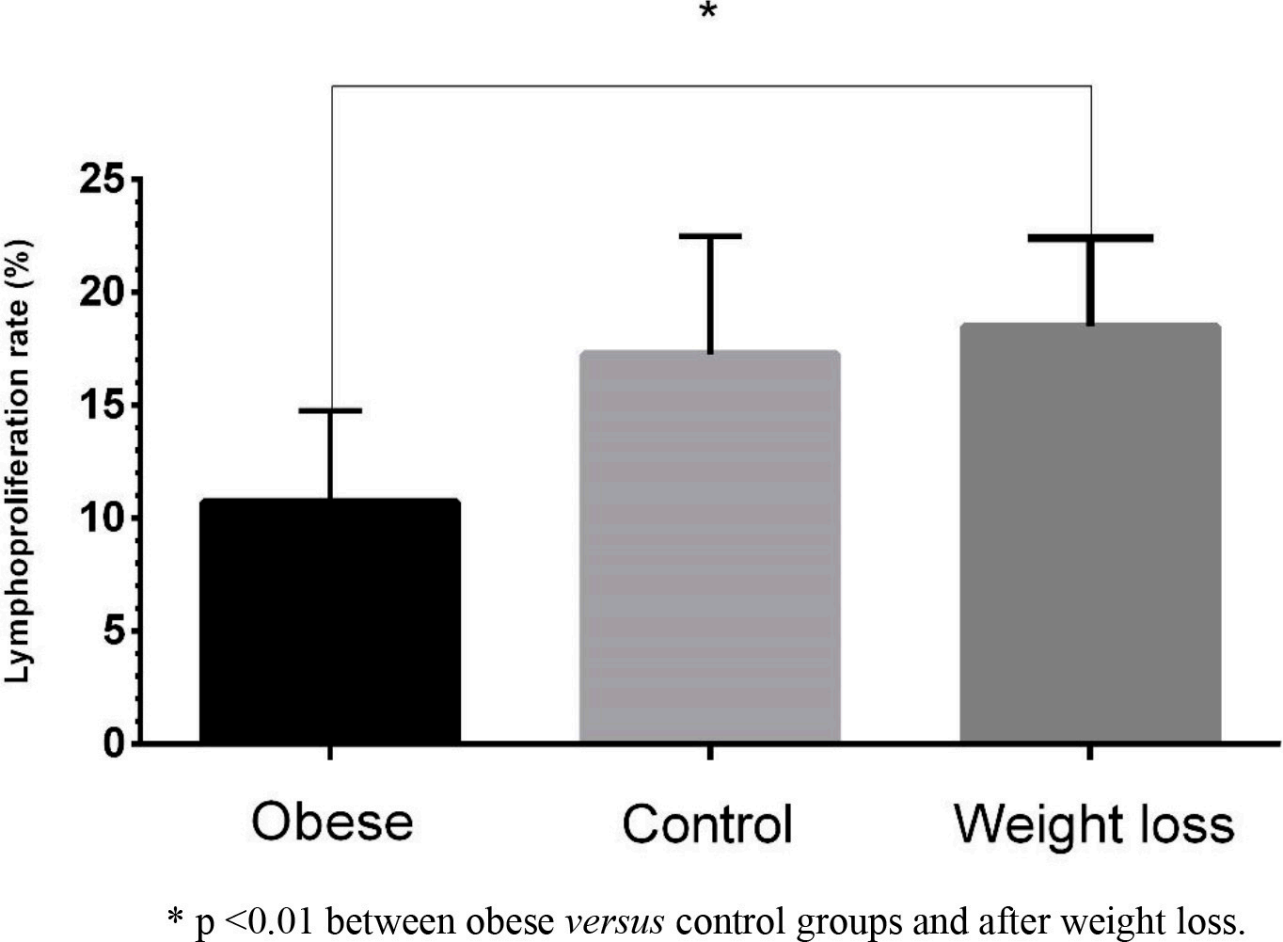
Lymphoproliferation ratio in the control group and the obese group before and after weight loss.

Lymphocyte proliferation was different between obese dogs before and after weight loss (P = 0.004; Fig 4), and weight loss resulted in an increase in lymphoproliferation ratio (18.48% vs 10.71% while obese). There was no difference in lymphoproliferation between the control group and the obese group after weight loss.

## Discussion

Throughout the study, the dogs that were included in the obese experimental group presented satisfactory food intake, which suggests good palatability of the diet despite low-fat content. The quality of the stool was adequate, according to the fecal score classification [52], and there were no reports of alterations during the period of the study. During the weight loss program, dogs were weighed and assessed every 15 days, and no clinical alterations were observed. Despite the ALT difference between the control group and the obese group after weight loss, all values for the biochemical analyses were considered in the species reference value range [53].

The mean weekly weight loss percentage was within the recommendation of 1 and 2% for dogs [54]. This result is important because it showed that the weight loss used in this study was efficient and promoted healthy weight loss. Most studies with client-owned dogs observed weekly weight loss ratio below 1% [35, 50, 55, 56], with ratios between 0.75 and 0.85%. The lower percentage of weight loss in client-owned animals may be explained by difficulty to comply with the weight loss program, providing more calories than expected by giving snacks [56]. All owners enrolled in the present study claimed that they followed protocol, thus the higher weekly weight loss ratio.

All animals included in the obese group had acquired and were obese for at least 2 years. This information is important to consider, as gene expression and immune alterations may not be observed when obesity is induced, especially if it occurs in the short term. Adipocytes undergo hyperplasia and hypertrophy to accommodate the increased demand of triglyceride intake due to overfeeding [39], and immune cells present in the adipose tissue have an important role during its remodeling, partly because of cytokine secretion, which facilitates vascular matrix remodeling necessary to healthy expansion of the adipose tissue [57]. The chronic expansion of the adipose tissue induces recruiting and excessive accumulation of immune cells and modulates innate (non-lymphocytes) and adaptive (lymphocyte) cells [58]. Furthermore, it also induces adipocyte transformation, including gene expression, intracellular proteases and adipokine secretion [59–61].

While transitioning from the lean to the obese state, adipocytes secrete small amounts of pro-inflammatory cytokines, but the circulating immune cells or those associated with the adipose tissue are responsible for the main secretion of these substances [62–66]. Adipokine production from the adipose tissue may be influenced by the nutritional status of the animal [67], in particular considering that obesity disrupts the balance of adipokine secretion, which is prejudicial to insulin action in peripheral tissues, such as muscle or liver [68]. In humans and rodents, the altered adipokine production and secretion in obesity have been related to various diseases, including diabetes mellitus, cardiovascular alterations, and cancer [69].

The increase in circulating resistin levels is considered to contribute to the development of insulin resistance and metabolic alterations compatible with those of type 2 diabetes mellitus in rats and humans [70–73], but little is known about its role in dogs. The results of the present study do not clearly indicate that the obese group before weight loss had insulin resistance, but lower resistin gene expression after weight loss suggests that obese animals are more prone to the action of this substance.

Studies regarding adiponectin observed that it negatively correlates to body fat in dogs [19, 74–78], but not in cats [79–81]. In the present study, adiponectin gene expression was higher in obese animals before weight loss than in the same group after weight loss, which is different from most studies. Doumatey et al. (2012) [82] evaluated the prevalence of paradoxical hyperadiponectinemia in obese humans, and its relation to metabolic healthy obesity phenotype. This phenotype was described by Karelis et al. (2008) [83] as obese individuals with metabolic profiles characterized by adequate sensitivity to insulin, and lipidic and hormonal profiles within reference ranges [83, 84]. Morrison et al. (2010) [85] also evaluated this paradox and concluded that increased serum adiponectin was associated with healthy obesity. Furthermore, another study [86] observed that approximately 20 to 30% of obese adults presented increased adiponectin concentrations. Therefore, despite controversial, in human medicine, there is a classification of “metabolically healthy yet obese” individuals [87]. A hypothesis that adiponectin was increased to counterbalance the effects of increase resistin and other pro-inflammatory cytokines was discussed by the authors of the present study, but further research is necessary to conclude on this matter.

Increased expression of IL-8 mRNA was observed in the obese group before weight loss when compared to the same group after weight loss, which corroborates with studies on humans. Recently, it was discovered that concentrations of IL-6 and MCP-1, but not of IL-8, were increased in overweight dogs [88]. Another study [89] observed that IL-8 concentrations decreased with weight loss in dogs. Vitger et al. (2017) [90] evaluated the glucose metabolism, cholesterol, adipokines, leptin, and adiponectin alterations, as well as inflammatory parameters such as C-reactive protein, IL-2, IL-6, IL-8, IL-10, MCP-1 in response to energy restriction with or without exercise. It was observed a decrease in IL-8 after weight loss only in the group that exercised. This is in correspondence with other studies in humans, in which exercise reduced plasmatic concentrations of similar parameters in obese individuals [91, 92]. In the present study, owners were encouraged to exercise their animals to help in the weight loss program, therefore the IL-8 increase observed may be due to exercise and not only due to weight loss.

Research in human medicine points to the link between increasing inflammatory mediators’ concentrations in obese individuals and the improvement in these parameters with weight loss [93, 94]. Recent research shows that IL-8, a neutrophil activator, may also be elevated in obese [95]. In humans, studies observed that IL-8 presented positive correlation with body mass index and increase of visceral fat mass [95, 96]. The chronic monocyte and macrophage accumulation in the adipose tissue contributes to increasing concentrations of pro-inflammatory signalment, being secreted along with adipocytes [96].

Regarding immune results, lymphocyte action is one of the main defense systems of the organism against invasion and proliferation of pathogens [97]. In the present study, the lymphoproliferation was evaluated by flow cytometry technique. In this technique, as the lymphocytes divide, half of the intracellular CFSE generates decreasing cell fluorescence peaks in a log scale, which is a more precise method to measure lymphoproliferation than traditional techniques [98, 99].

Results of the present study indicate that after weight loss animals presented lymphoproliferation rates similar to animals with ideal BCS, which means that the immune deficit caused by obesity can be reversed after an effective weight loss program. It is important to state that obesity in humans and rats has been associated with immune alterations and low-grade inflammation status [100–104]. Data obtained by Takamura et al. (2007) [103] in rats corroborate that reduction of body fat in obese animals improves lymphoproliferation characteristics. According to Greeley et al. (2006) [105], mild energy restriction and maintenance of adequate BCS may delay immune cell function decline related to ageing in dogs.

Van de Velde et al. (2012) [106] observed that weight gain and increased BCS were followed by higher concentrations of leptin, IgA, IgM and lymphocyte function, as well as an increased response to in vitro mitogenic stimulation of mononuclear cells from peripheral blood. However, when the immune response was evaluated in a stable obese condition, no immune function alterations or low-grade inflammation were observed [107]. Although no alterations in the immune function were observed at the end of the study, the fact that obesity was induced in a period of 47 weeks, followed by a period of 26 weeks of stable body weight, may have influenced results. The authors observed an increase in T lymphocytes after the weight gain period, but this increase was transitory and after weight stabilization no alteration was observed [107].

Results from the present study, along with results observed by Van de Velde et al. (2013) [107] suggest that change in the energy balance during the process of weight gain (becoming obese vs. being obese) may impair lymphocyte function, and can be corrected by weight stabilization and, even more importantly, by weight loss.
